# Adeno-Associated Virus as an Effective Malaria Booster Vaccine Following Adenovirus Priming

**DOI:** 10.3389/fimmu.2019.00730

**Published:** 2019-04-05

**Authors:** Yenni Yusuf, Tatsuya Yoshii, Mitsuhiro Iyori, Kunitaka Yoshida, Hiroaki Mizukami, Shinya Fukumoto, Daisuke S. Yamamoto, Asrar Alam, Talha Bin Emran, Fitri Amelia, Ashekul Islam, Hiromu Otsuka, Eizo Takashima, Takafumi Tsuboi, Shigeto Yoshida

**Affiliations:** ^1^Laboratory of Vaccinology and Applied Immunology, Kanazawa University School of Pharmacy, Kanazawa University, Kanazawa, Japan; ^2^Department of Parasitology, Faculty of Medicine, University of Hasanuddin, Makassar, Indonesia; ^3^Kanazawa University Graduate School of Medical Sciences, Kanazawa University, Kanazawa, Japan; ^4^Division of Gene therapy, Jichi Medical University, Shimotsuke, Japan; ^5^National Research Centre for Protozoan Diseases, Obihiro University of Agriculture and Veterinary Medicine, Obihiro, Japan; ^6^Division of Medical Zoology, Department of Infection and Immunity, Jichi Medical University, Shimotsuke, Japan; ^7^Division of Malaria Research, Proteo-Science Center, Ehime University, Matsuyama, Japan

**Keywords:** *Plasmodium falciparum* circumsporozoite protein, Pfs25, human adenovirus serotype 5, adeno-associated virus, malaria vaccine, transmission-blocking

## Abstract

An ideal malaria vaccine platform should potently induce protective immune responses and block parasite transmission from mosquito to human, and it should maintain these effects for an extended period. Here, we have focused on vaccine development based on adeno-associated virus serotype 1 (AAV1), a viral vector widely studied in the field of clinical gene therapy that is able to induce long-term transgene expression without causing toxicity *in vivo*. Our results show the potential utility of AAV1 vectors as an extremely potent booster vaccine to induce durable immunity when combined with an adenovirus-priming vaccine in a rodent malaria model. We generated a series of recombinant AAV1s and human adenovirus type 5 (AdHu5) expressing either *Plasmodium falciparum* circumsporozoite protein (PfCSP) or P25 (Pfs25) protein. Heterologous two-dose immunization with an AdHu5-prime and AAV1-boost (AdHu5-AAV1) elicited robust and durable PfCSP- or Pfs25-specific functional antibodies over 280 days. Regarding protective efficacy, AdHu5-AAV1 PfCSP achieved high sterile protection (up to 80% protection rate) against challenge with transgenic *Plasmodium berghei* sporozoites expressing PfCSP. When examining transmission-blocking (TB) efficacy, we found that immunization with AdHu5-AAV1 Pfs25 maintained TB activity *in vivo* against transgenic *P. berghei* expressing Pfs25 for 287 days (99% reduction in oocyst intensity, 85% reduction in oocyst prevalence). Our data indicate that AAV1-based malaria vaccines can confer potent and durable protection as well as TB efficacy when administered following an AdHu5 priming vaccine, supporting the further evaluation of this regimen in clinical trials as a next-generation malaria vaccine platform.

## Introduction

In response to the threat posed by emerging resistance to artemisinin-based chemotherapy and insecticide-treated bed nets, efforts to develop potent malaria vaccines as a complementary tool in eradicating the disease have recently been intensified (1). The most advanced *Plasmodium falciparum* malaria vaccine candidate, RTS,S/AS01 (also known as Mosquirix™), which is directed against the pre-erythrocytic stage and targets the *P. falciparum* circumsporozoite protein (PfCSP), showed a limited vaccine efficacy of 36.3% against clinical malaria in phase III clinical trials in several sub-Saharan countries; however, this efficacy declined to 4.4% over 7 years of follow-up (2, 3). In addition to its limited and short-term efficacy, RTS,S raised some safety concerns and practical deployment challenges with its four-dose regimen in target age groups at high risk of malaria (4). Because of the moderate efficacy of the RTS,S vaccine, the Malaria Vaccine Roadmap has updated their strategic goals from the development of vaccines with 80% protective efficacy against *P. falciparum* by 2020 to the development of second-generation malaria vaccines for malaria elimination in multiple settings that are highly efficacious against the disease by 2030 (1, 5). Achieving this goal would require current control strategies to be complemented by new breakthroughs in vaccine development.

A promising strategy for blocking transmission of the parasite from mosquitoes to humans is the development of transmission-blocking (TB) vaccines (TBVs) targeting the antigens expressed in the sexual stages of malarial parasites. However, one potential limitation of TBVs is their restricted activity; because levels of specific antibody against the antigen, particularly the mosquito-stage antigen Pfs25, cannot be boosted by natural infection, the titer gradually falls over time (6, 7). Hence, the development of a TBV capable of inducing long-term TB immunity for at least one transmission season (~6 months), to be combined with effective pre-erythrocytic stage vaccines, would be an advantageous strategy (6).

Adeno-associated virus (AAV), a member of the family *Parvoviridae*, can infect a wide variety of human and nonhuman cells and is not associated with any known disease or adverse clinical effects. A key advantage of AAV vectors is their capability to mediate long-term transgene expression without causing toxicity *in vivo* (8, 9). Its safety and durability profiles have made AAV an attractive vector for gene therapy, and it has been tested in around 100 clinical trials (10, 11). Recently, AAV vectors have also emerged as the frontrunner in vectored immunoprophylaxis (VIP), an active approach to substitute passive immunization that acts by facilitating the host secretion of neutralizing antibodies following the delivery by AAV of genes encoding these antibodies (12, 13). Intramuscular (i.m.) injection of VIP vectors in mice and macaques elicits long-lived antibody or antibody-related immunoadhesin production at levels sufficient to protect against HIV, simian immunodeficiency virus, and influenza A virus infection (14–17). Because both VIP and gene therapy rely on the low immunogenicity of AAV to permit durable expression of the transgene, utilization of AAV in the field of immunization is minimal (18). Of its few investigated applications, an AAV-based malaria blood-stage vaccine has been developed, but this vaccine did not confer any protection against malaria parasite challenge in a mouse model, either when used in a single vaccine regimen or as a booster following a prime with DNA or another AAV serotype (19, 20).

The present study aimed to develop a potent malaria vaccine platform to effectively induce durable immunity for both protection and TB. In pursuit of this aim, we investigated the potential efficacy of AAV-vectored vaccines harboring either the *pfcsp* or *pfs25* gene by applying a heterologous prime-boost immunization regimen with other viral-vectored or protein-in-adjuvant vaccines. We used transgenic *P. berghei* expressing either the *pfcsp* or *pfs25* genes in a murine model to evaluate the protective and TB efficacies of these regimens.

## Materials and Methods

### Parasites and Animals

Transgenic *P. berghei* Pfs25DR3, which was used for the TB assays, was kindly donated by Andrew Blagborough from Imperial College London (21). Transgenic *P. berghei* expressing PfCSP (PfCSP-Tc/Pb) for the protective efficacy study was described previously (21–23). Both transgenic parasites were maintained in the Laboratory of Vaccinology and Applied Immunology, Kanazawa University. *Anopheles stephensi* mosquitoes (SDA 500 strain) were infected with the transgenic parasites by allowing them to feed on parasite-infected 6-week-old ddY mice. All other animal experiments used 6-week-old BALB/c mice.

### *In vivo* Bioluminescent Imaging

AAV1 expressing luciferase (AAV1-Luc) was administered into the right medial thigh muscles of BALB/c mice (*n* = 3; 1 × 10^11^ viral genomes [vg]/mouse) on day 0, and D-Luciferin (15 mg/mL; OZ Biosciences, Marseille, France) was administered intraperitoneally (i.p.; 150 μL/mouse) at the appropriate timepoints. Luciferase expression was detected as described previously (23, 24). The accumulated emissions were calculated, and their intensities are expressed in a color heat map.

### Viral Vector Construction

To generate AAV1-PfCSP-G(–) and AAV1-PfCSP-G(+), the gene cassette encoding the mouse IgGκ signal peptide, FLAG tag, and WPRE was first synthesized and cloned into pUC57-Simple, to construct pUC57-Simple-SP-FLAG-WPRE (GenScript, Piscataway, NJ, USA) (Figure S1). The codon-optimized gene encoding a GPI anchor-lacking PfCSP (Leu_19_-Val_377_) was excised from pENTR-CAG-sPfCSP2-G2-sWPRE by digestion with EcoRI and XmaI and then inserted into the MunI and XmaI sites of pUC57-Simple-SP-FLAG-WPRE to construct pUC57-sPfCSP2-WPRE. Next, the gene cassette encoding SP-FLAG-sPfCSP2-WPRE was excised from pUC57-sPfCSP2-WPRE by digestion with EcoRI and XhoI and then inserted into the EcoRI and XhoI sites of pAAV-MCS under the control of the CMV promoter sequence to construct pAAV-CMV-sPfCSP2-G(–). The gene encoding PfCSP with VSV-G was excised from pENTR-CAG-sPfCSP2-G2-sWPRE by digestion with BamHI and XhoI and then inserted into the BamHI and XhoI sites of pAAV-CMV-sPfCSP2-G(–) to construct pAAV-CMV-sPfCSP2-G(+). These plasmids, pAAV-CMV-sPfCSP2-G(–) and pAAV-CMV-sPfCSP2-G(+), were used to produce the AAV1-PfCSP-G(–) and AAV1-PfCSP-G(+), respectively, in HEK293 cells as described elsewhere (8).

To generate AdHu5-Pfs25, the codon-optimized genes encoding Pfs25 and G6S hinge were first synthesized and cloned into pUC57-Simple to construct pUC57-Simple-sPfs25-hinge (GenScript) (Figure S1). The *pfs25* gene fragment was then excised from pUC57-Simple-sPfs25-hinge by digestion with EcoRI and AgeI and inserted into the EcoRI and XmaI sites of pENTR-CAG-sPfCSP2-G2-sWPRE to construct pENTR-CAG-sPfs25-G2-sWPRE. This plasmid, pENTR-CAG-sPfs25-G2-sWPRE, was cloned into the shuttle vector pAd/PL-DEST (Invitrogen, Carlsbad, CA, USA) using the LR recombination reaction. The resulting adenovirus was purified and titrated using the Fast-Trap Adenovirus Purification and Concentration Kit (Millipore, Temecula, CA, USA) and the Adeno-X™ Rapid Titer Kit (Clontech, Palo Alto, CA, USA), respectively, according to the manufacturers' protocols. AdHu5-PfCSP has been described previously (25).

To generate AAV1-Pfs25, the gene cassette encoding SP-FLAG-sPfs25-WPRE was first excised from pENTR-CAG-sPfs25-G2-sWPRE by digestion with EcoRI and XhoI and then inserted into the EcoRI and XhoI sites of pAAV-MCS under the control of the CMV promoter sequence, to construct pAAV-CMV-Pfs25. This plasmid, pAAV-CMV-Pfs25, was subsequently used to produce AAV1-Pfs25 in HEK293 cells as described elsewhere (8).

### Immunoblotting

HEK293T cells were transduced with the adenoviral vaccines at a multiplicity of infection (MOI) of 3 or 10 or with the AAV1 vaccines at a MOI of 10^5^ or 10^6^ at 24 h after being seeded into plates. Cell lysates were collected using Laemmli buffer at 48 h post-transduction and subjected to immunoblotting. The cell lysates were electrophoresed on 10% sodium dodecyl sulfate polyacrylamide (SDS-PAGE) gels under reducing conditions for PfCSP and under non-reducing conditions for Pfs25. Samples were transferred electrophoretically onto an Immobilon FL^®^ PVDF membrane (Merck Millipore, Tokyo, Japan). The membranes were blocked for up to 1 h using 5% skim milk in PBS containing 0.1% Tween 20 (PBS-T), then incubated for 1 h at room temperature with the monoclonal antibody (mAb) anti-PfCSP 2A10 or mAb anti-Pfs25 4B7, diluted 1:10,000 in 5% skim milk. After being washed with PBS-T, the blots were probed with the secondary antibody, goat anti-mouse conjugated to IRDye 800 (Rockland Immunochemicals, Limerick, PA, USA), diluted 1:20,000 in 5% skim milk. The membrane was then visualized using an Odyssey infrared imager (LI-COR, Lincoln, NE, USA). The molecular weight predictions were performed using the ExPASy server.

### Immunofluorescence Assay (IFA)

HEK293T cells grown on eight-well chamber slides were transduced with the adenoviral vaccines or AAV1 vaccines at a MOI of 10 or 10^5^, respectively. The cells were fixed with 100% methanol or 4% paraformaldehyde for 30 min at 24 h post-transduction. After being washed with PBS, the slides were blocked for 1 h with 10% normal goat serum in PBS. The cells were then incubated for 1 h at room temperature with Alexa-Fluor-488-conjugated 2A10 or Alexa-Fluor-596-conjugated 4B7, diluted 1:100 in blocking solution. After being washed with PBS, the slide was mounted with a drop of VECTASHIELD containing 4′,6-diamidino-2-phenylindole (DAPI; Vector Laboratories, Burlingame, CA, USA). A BZ-X710 fluorescence microscope (Keyence Corp, Tokyo, Japan) was used for image acquisition.

### Immunization

All vaccines were administered intramuscularly in 100 μL of endotoxin-free PBS. Adenoviral vaccines were administered at a dose of 5 × 10^7^ plaque-forming units (PFU), while AAV1 vaccines were administered at a dose of 10^11^ vg per mouse. Insect baculovirus expressing PfCSP (BV-PfCSP) was administered at a dose of 10^8^ PFU, and recombinant full-length PfCSP (rPfCSP) was administered at a dose of 10 μg in Imject® Alum (Thermo Scientific, Waltham, MA, USA).

### ELISA

PfCSP- or Pfs25-specific antibody levels were quantified by ELISA as described previously (23). The Pfs25 antigen, constructed using the same *pfs25* gene as in the viral-vectored vaccines, was produced using the wheat germ cell-free (WGCF) protein expression system (CellFree Sciences, Matsuyama, Japan) (26). Sera from immunized mice were collected from tail vein blood samples 1 day before boost and 1 day before challenge or weekly up to 280 days post-boost for monitoring. Costar® EIA/RIA polystyrene plates (Corning Inc, NY, USA) precoated with 400 ng/well of PfCSP or 200 ng/well of Pfs25 were blocked with 1% bovine serum albumin (BSA) in PBS and then incubated with serially diluted sera samples, as well as with negative and positive controls (mAb 2A10 or mAb 4B7, respectively). An anti-mouse IgG antibody conjugated with horseradish peroxidase (HRP) (Bio-Rad lab, Inc, Tokyo, Japan) was used as the secondary antibody. Endpoint titers are expressed as the reciprocal of the last dilution that gave an optical density at 414 nm of 0.15 U above the values of the negative controls (<0.1). All mice used in our experiments were seronegative before immunization.

### Intracellular Cytokine Staining (ICS) and *ex vivo* Interferon (IFN)-γ Enzyme-Linked Immune Absorbent Spot (ELISpot) Assay

ICS and ELISpot were performed using splenocytes as described previously (25). For ICS, the splenocytes were stimulated for 6 h with a final concentration of 1 μg/mL of the immunodominant CD8^+^ T-cell epitope NYDNAGTNL (PfCSP_39−47_) and 1 μg/mL of GolgiPlug™ (BD Biosciences, Tokyo, Japan) in a 96-well U-bottom tissue culture plate (Corning Inc.). The cells were then surface stained with anti-mouse CD16/32 antibody, Pacific Blue™-conjugated anti-mouse CD4 antibody, and PerCP/Cy5.5-conjugated anti-mouse CD8β antibody, and the cytokine was stained with fluorescein isothiocyanate (FITC)-conjugated anti-mouse IFN-γ antibody or a FITC-conjugated rat IgG1κ isotype control antibody. Data were acquired with a BD FACSVerse™ Flow Cytometer (BD Biosciences) and analyzed with FlowJo (Tree Star, Ashland, OR, USA). All antibodies used in these experiments were purchased from BioLegend (San Diego, CA, USA).

For ELISpot assays, splenocytes were cultured for 20–24 h on an ELISpot microplate (PerkinElmer, Yokohama, Japan) with the *H-2K*^*d*^-restricted PfCSP T-cell epitope (NYDNAGTNL, PfCSP_39−47_; final concentration, 1 μg/mL) or the PfCSP-overlapping peptide pool (final concentration, 5 μg/mL). Results are expressed as IFN-γ spot-forming units (SFU) per million splenocytes.

### Parasite Challenge Test

Mice were intravenously challenged with PfCSP-Tc/Pb sporozoites resuspended in RPMI-1640 media (Gibco, Life Technologies, Tokyo, Japan). Sporozoites were prepared as described previously (27). Each mouse was injected with 100 μL of media containing 1,000 or 500 sporozoites via the tail vein. Infection was monitored from day 4 to 14 by Giemsa staining of thin blood smears using blood samples obtained from the tail. Protection was defined as the complete absence of blood-stage parasitemia on day 14 post-challenge. Protective efficacy was calculated using the following formula: % protective efficacy = {1 – [(number of infected mice in the vaccine group/total number of mice in the vaccine group)/(number of infected mice in the non-immunized group/total number of mice in the non-immunized group)]} × 100.

### TB Assays

TB was assessed using direct-feeding assays (DFAs). At 35 or 287 days after boost, the mice were treated with phenylhydrazine (PHZ) and then infected i.p. with 10^6^ *P. berghei* Pfs25DR3-parasitized red blood cells (pRBCs) 3 days later. At 3 days post-infection, at least 50 starved *A. stephensi* mosquitoes were allowed to feed on each infected mouse. At 5–6 h post-feeding, any unfed mosquitoes were removed. Mosquitoes were then maintained on fructose [8% (w/v) fructose, 0.05% (w/v) *p*-aminobenzoic acid] at 19–22°C and 50–80% relative humidity. On day 10–12 post-feeding, the mosquito midguts were dissected, and oocyst prevalence and intensity were recorded. For each mouse, the number of oocysts was counted, and the mean oocyst intensity was calculated. For inhibition calculations, these numbers were compared with those of mice immunized with AdHu5-AAV1-Luc control. Percent (%) inhibition of mean oocyst intensity (transmission-reducing activity; TRA) was calculated as follows: 100 × [1 – (mean number of oocysts in the test group/mean number of oocysts in the control group)]. Similarly, the % inhibition of oocyst prevalence (transmission-blocking activity; TBA) was evaluated as: 100 × [1 – (proportion of mosquitoes with any oocysts in the test group)/(proportion of mosquitoes with any oocysts in the control group)] (28).

### Statistical Analysis

For all statistical analyses, GraphPad Prism version 7.0 for Mac OS was used. Depending on the data distribution, a Student's *t*-test, Mann–Whitney rank test, or Wilcoxon matched-pairs signed rank test was used for comparing two groups. For the analysis of differences among immunization groups, a Kruskal–Wallis test with Dunn's correction for multiple comparisons or Tukey's multiple comparison was used. All ELISA end-point titers were log_10_ transformed before analysis. The protection level was analyzed by a Fisher's exact test. The significance of TRA and TBA was assessed using the Mann–Whitney *U*-test and Fisher's exact probability test, respectively. A *p* < 0.05 was considered statistically significant.

## Results

### Durable Luciferase Expression by AAV1-Luc at the Injection Site

**Figure 1:**
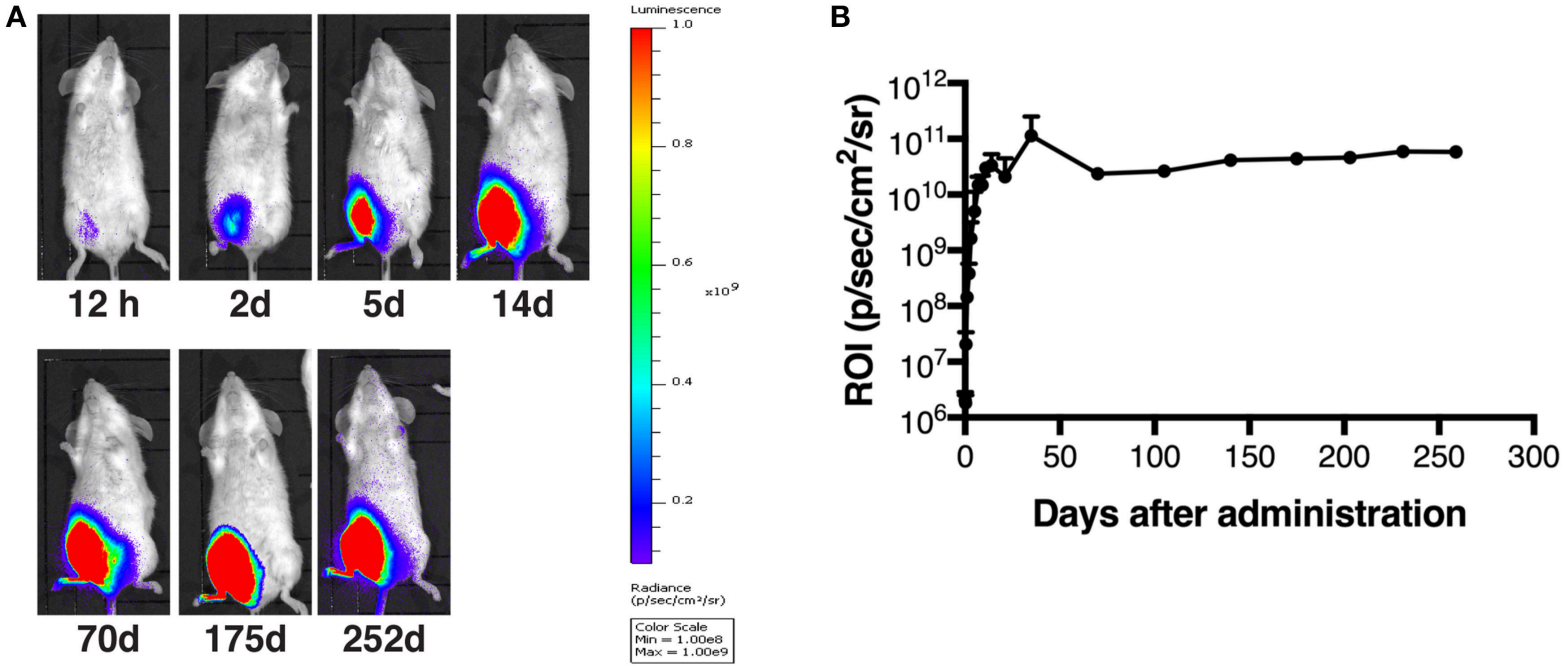
Long-term transgene expression after muscle transduction with AAV1-Luc. **(A)** Luciferase expression at different timepoints, detected by using the IVIS Lumina LT Series III *in vivo* imaging system. AAV1-Luc was administered into the right medial thigh muscles of BALB/c mice (*n* = 3; 1 × 10^11^ vg/mouse) on day 0. Luciferase expression remained high up to 252 days post-administration of AAV1-Luc. The heat map images visible in the mice represent the total flux of photons (p/s/cm^2^) in that area. Rainbow scales are expressed in radiance (p/s/cm^2^/sr). **(B)** The mean total flux of photons is shown as a region of interest (ROI) from day 0 to day 252 after administration of AAV1-Luc.

It has been shown that AAV vectors are capable of expressing a transgene for a long time and of transducing skeletal muscle efficiently while inducing minimal inflammatory responses (8, 29). To examine the transduction efficacy and durable transgene expression of our AAV1, AAV1-Luc was administered into the right medial thigh muscles of BALB/c mice (10^11^ vg/mouse; *n* = 3) on day 0. Luciferase expression was monitored with bioluminescence imaging (Figure 1A), and the data for the total flux (Figure 1B) at different timepoints were normalized against that at day 1. The luminescence signal increased gradually from day 0 to day 7, reaching the peak within 10 days (10^10^ p/s/cm^2^/sr); as expected, robust luciferase expression persisted for up to 252 days (Figures 1A,B). This result indicates that our AAV1 vector system can efficiently transduce muscle cells and achieve durable expression of the transgene product in mouse muscle, consistent with other studies showing a high level of stable transgene expression after an i.m. injection of AAV serotype 1 or 2, lasting for 1–5 years (30–32).

### Construction of the AAV1-PfCSP-G(–) Vaccine

**Figure 2:**
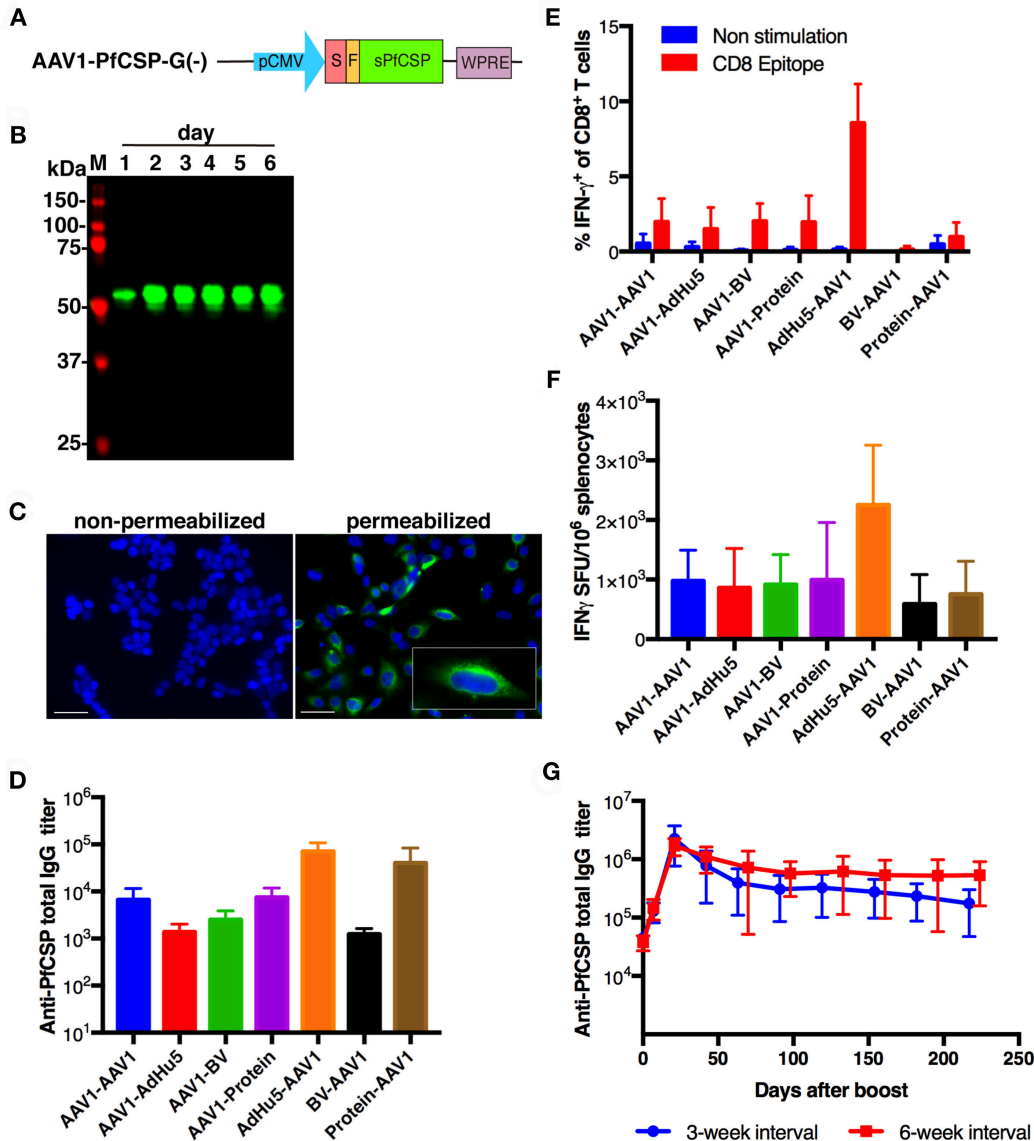
Functional activity of AAV1-PfCSP-G(–). **(A)** Construction of AAV1-PfCSP-G(–). Expression of the *pfcsp* gene cassette was driven by the CMV promoter. S, signal sequence; F, FLAG epitope tag. **(B)** Expression of PfCSP in HEK293T cells transduced with AAV1-PfCSP-G(–) (MOI = 10^5^), as assessed by immunoblotting with anti-PfCSP mAb 2A10. M, molecular marker. **(C)** Localization of PfCSP expression in transduced cells. HEK293T cells were transduced with AAV1-PfCSP-G(–) (MOI = 10^5^) as determined by IFA. After 48 h, cells were fixed with methanol (permeabilized) or paraformaldehyde (non-permeabilized) and incubated with Alexa-Fluor-488-conjugated mAb 2A10 (green). Cell nuclei were visualized with 4′,6-diamidino-2-phenylindole (DAPI; blue). Original magnification, ×400. Bars = 50 μm. **(D)** Anti-PfCSP IgG antibody responses. BALB/c mice (*n* = 3) were immunized with the indicated regimens at a 3-week interval. Two weeks after boosting, serum samples were collected from each mouse, and their anti-PfCSP IgG titers were determined by ELISA. AdHu5-PfCSP, BV-PfCSP, AAV1-PfCSP-G(–), and rPfCSP protein are shown as AdHu5, BV, AAV1, and protein, respectively. Bars and error bars indicate the means and SD of the values, respectively. **(E,F)** PfCSP-specific cellular immune responses. BALB/c mice were immunized as described in **(D)**. At 2 weeks post-boost, splenocytes were stimulated with the synthetic PfCSP-specific CD8^+^ T-cell epitope. **(E)** An ICS assay was performed on the splenocytes. Percentages of IFN-γ-secreting cells in the CD8^+^CD4^−^ T-cell population are shown after the subtraction of the percentages of cells stained with an isotype control antibody. **(F)** An *ex vivo* ELISpot assay was performed on splenocytes from the same mice. The IFN-γ SFU that reacted with the PfCSP-specific CD8^+^ T-cell epitope per million splenocytes are shown. **(G)** Monitoring of anti-PfCSP IgG antibody responses. Groups of BALB/c mice (*n* = 5) were immunized with an AdHu5-PfCSP -prime and AAV1-PfCSP-G(–)-boost regimen at a 3- or 6-week interval. Serum samples were collected from each mouse 1 day before boost and weekly after boost. Anti-PfCSP IgG titers were determined by ELISA and monitored for 224 days after booster injection.

For the construction of pre-erythrocytic-stage vaccines, we generated AAV1-PfCSP-G(–) harboring a gene cassette encoding GPI anchor-lacking PfCSP (Leu_19_-Val_377_), followed by a *wpre* sequence, under the control of the CMV immediate-early enhancer-promoter (Figure 2A). AAV1-PfCSP-G(–) was designed to allow PfCSP to be secreted from transduced cells, which is a construction similar to that used in VIP. Immunoblotting revealed that the expression level of PfCSP in HEK293 cells increased gradually until 6 days after transduction without any cytopathic effect (Figure 2B; Figure S2); this is consistent with the expression pattern of luciferase shown in Figure 1. An IFA analysis showed that PfCSP was accumulated in the cytoplasm but not on the surface of transduced cells (Figure 2C). Unexpectedly, no trace of PfCSP expression was detected in the cell medium by either immunoprecipitation or ELISA (data not shown). These data indicate that PfCSP was not secreted from cells transduced with AAV1-PfCSP-G(–). A similar phenomenon in which full-length PfCSP was expressed in only the cytoplasm of insect cells has been reported previously (33). The secretion seems to be highly dependent on amino acid sequences and structure of proteins.

### Boosting With AAV1-PfCSP-G(–) Following AdHu5-PfCSP Priming Induces Potent and Durable Anti-PfCSP Immune Responses

We have previously generated rPfCSP by an *Escherichia coli* expression system (22, 23, 25, 34) as well as AdHu5-PfCSP and BV-PfCSP (25). To determine the optimal regimen of AAV1 immunization, we investigated several heterologous prime-boost immunization regimens using AAV1-PfCSP-G(–) in combination with these vaccines, in addition to the homologous AAV1, and we compared the induction of PfCSP-specific humoral and cellular immune responses. Mice were immunized with a 3-week interval between the prime and boost (*n* = 3). At 2 weeks post-boost, sera were collected from these mice for ELISAs, and their splenocytes were isolated for ICS and ELISpot assays. The highest anti-PfCSP IgG titer was induced by the AdHu5-PfCSP-prime/AAV1-PfCSP-G(–)-boost heterologous regimen (AdHu5-AAV1 PfCSP) (Figure 2D). ICS assays revealed that AdHu5-AAV1 PfCSP induced the highest IFN-γ production by CD8^+^ T cells after stimulation with the CSP-derived *H-2K*^*d*^ peptide (Figure 2E). ELISpot assays also showed that AdHu5-AAV1 PfCSP induced the highest frequency of IFN-γ-secreting cells, with an average of 2,252.22 SFU compared with <1,000 SFU per million splenocytes induced by other regimens (Figure 2F). Thus, although these experiments were not powered to detect statistically significant differences in immune responses, the cellular immune responses demonstrated the same trend as seen in the humoral responses. Accordingly, we used the AdHu5-AAV1 regimen for further vaccine evaluation. Next, to optimize the AdHu5-AAV1 immunization regimen, two different intervals between prime and boost, 3- and 6-week, were compared by evaluating the kinetics of the antibody responses to PfCSP. Both regimens induced the highest anti-PfCSP IgG titers (1–2 × 10^6^) at day 21 after boost and persisted at a high level (>10^5^) for 224 days (Figure 2G). We found that the 6-week interval regimen tended to maintain higher levels of anti-PfCSP IgG titers at some points compared with the 3-week interval regimen. Therefore, the 6-week interval regimen was used for all further experiments.

### AAV1-PfCSP-G(+) Is Much Superior to AAV1-PfCSP-G(–)

To assess the protective efficacy of heterologous prime-boost regimens using AdHu5-PfCSP and AAV1-PfCSP-G(–), immunized mice were challenged with PfCSP-Tc/Pb sporozoites 2 weeks post-boost, and the presence of blood infection was evaluated up to 14 days post-challenge. For comparison, we also performed challenges with homologous AdHu5-PfCSP (AdHu5-AdHu5), homologous AAV1-PfCSP-G(–) (AAV1-AAV1), and heterologous prime-boost of AAV1-PfCSP-G(–)-prime and AdHu5-PfCSP-boost (AAV1-AdHu5). The AdHu5-AAV1 immunization regimen conferred only a moderate sterile protection rate (37.5%), but this rate was the highest among the four tested regimens (Table 1, experiment 1).

**Table 1:** Protective efficacies of heterologous prime and boost regimens using AdHu5 and AAV1 vaccines against sporozoite challenge^a^.

Prime	Boost	Protected/challenged (% protective efficacy^b^)
**EXPERIMENT 1**		
PBS	PBS	2/10 (0)
AAV1-G(–)	AAV1-G(–)	2/10 (0)
AAV1-G(–)	AdHu5	2/10 (0)
AdHu5	AAV1-G(–)	5/10 (37.5)
AdHu5	AdHu5	3/10 (12.5)
**EXPERIMENT 2**		
PBS	PBS	0/10 (0)
AdHu5	AAV1-G(–)	2/10 (20)
AdHu5	AAV1-G(+)	8/10 (80)^c^

a *AdHu5-PfCSP, AAV1-PfCSP-G(–), and AAV1-PfCSP-G(+) are shown as AdHu5, AAV1-G(–), and AAV1-G(+), respectively. Immunized mice were intravenously challenged with 1,000 (Exp. 1) or 500 (Exp. 2) PfCSP-Tc/Pb sporozoites and checked for blood-stage infections by microscopic examination of Giemsa-stained thin smears of tail blood. Protection was defined as the complete absence of blood-stage parasitemia on day 14 post-challenge*.

b *Protective efficacy was calculated as described in the Materials and Methods*.

c *Significant difference with the PBS group as determined using a Fisher's exact probability test (p < 0.001)*.

**Figure 3:**
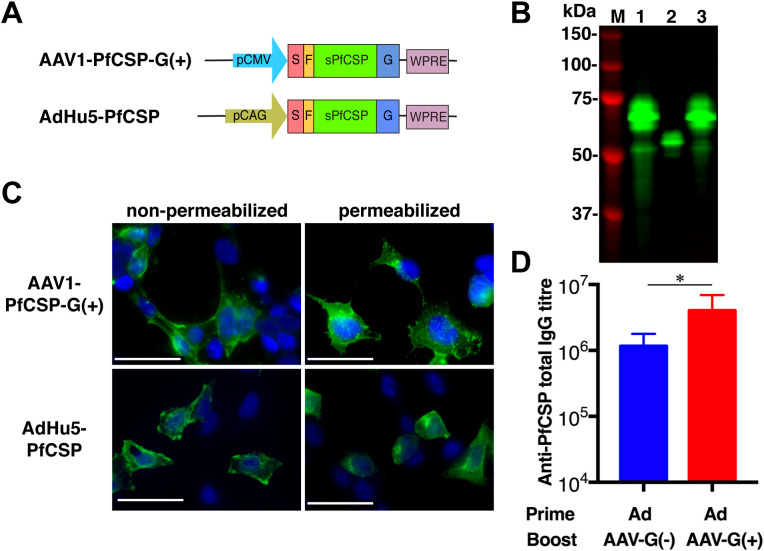
Functional activity of AAV1-PfCSP-G(+). **(A)** Constructs of AAV1-PfCSP-G(+) and AdHu5-PfCSP-G(+). Expression of the *pcsp* gene cassette in AAV1 and AdHu5 was driven by a CMV promoter and CAG promoter, respectively. G, VSV-G. **(B)** Expression of PfCSP in HEK293T cells transduced with AdHu5-PfCSP (lane 1, MOI = 3), AAV1-PfCSP-G(–) (lane 2, MOI = 10^5^), or AAV1-PfCSP-G(+) (lane 3, MOI = 10^5^), as assessed by immunoblotting with mAb 2A10 at 48 h post-transduction. **(C)** Localization of PfCSP expression in HEK293T cells transduced with AAV1-PfCSP-G(+) (MOI = 10^5^) and AdHu5-PfCSP (MOI = 10), as determined by IFA conducted as described in Figure 2C. **(D)** Anti-PfCSP IgG antibody responses. Groups of BALB/c mice (*n* = 10) were immunized with the indicated regimen at a 6-week interval. At 4 weeks post-boost, serum samples were collected from each mouse, and their anti-PfCSP IgG titers were determined by ELISA. AdHu5-PfCSP, AAV1-PfCSP-G(–), and AAV1-PfCSP (G+) are shown as AdHu5, AAV1-G(–), and AAV1-G(+), respectively. Bars and error bars indicate the means and SD of the values, respectively. Between-group differences were assessed with a Mann–Whitney *U*-test (^*^p < 0.05).

In an effort to improve the protective efficacy, we generated AAV1-PfCSP-G(+), which anchors PfCSP on the surface of infected cells via the VSV-G protein membrane anchor (Figures 3A,C). The glycoprotein G of VSV is a 70-kDa glycoprotein containing two asparagine-linked complex oligosaccharides and is positioned such that almost 90% of the polypeptide chain is external to the lipid bilayer, forming spikes on the surface of the virion (35, 36). Thus, fusing an antigen to VSV-G allows a more efficient display of the antigen on infected cells (35). Transduction by AAV1-PfCSP-G(+) resulted in the same expression pattern on the cell surface as transduction by AdHu5-PfCSP (25). Immunoblotting with a quantification analysis showed that the amount of PfCSP expressed following transduction by AAV1-PfCSP-G(+) at MOI = 10^5^ (PfCSP-VSV-G: predicted *Mr* of 53.3 kDa;) was three times higher than that following transduction by AAV1-G(–) (PfCSP: predicted *Mr* of 43.9 kDa) (Figure 3B, lanes 3 and 2), which was similar to the amount of PfCSP induced by AdHu5-PfCSP (MOI = 3, lane 1). Importantly, boosting with AAV1-G(+) following an AdHu5-prime evoked significantly higher anti-PfCSP IgG titers than did boosting with AAV1-G(–) (1.15 × 10^6^ vs. 4.03 × 10^6^, *p* < 0.05) (Figure 3D). This result indicates that anchoring PfCSP through VSV-G enhanced not only the PfCSP expression level but also the induced humoral immune responses.

We then compared the boosting effects of AAV1-PfCSP-G(–) and AAV1-PfCSP-G(+) on protective efficacy. Mice immunized with a boost of either AAV1-PfCSP-G(–) or AAV1-PfCSP-G(+) following an AdHu5-PfCSP prime at a 6-week interval were challenged with PfCSP-Tc/Pb sporozoites 5 weeks after boost (*n* = 10/group). A significantly higher level of sterile immunity was achieved by AAV1-G(+) (80%) than by AAV1-G(–) (20%) (Table 1, Experiment 2). These results indicate that the display of PfCSP on the infected cells effectively enhanced the protective efficacy with the induction of a higher anti-PfCSP antibody response.

### Construction and Expression of the AAV1-Pfs25 and AdHu5-Pfs25 Vaccines

**Figure 4:**
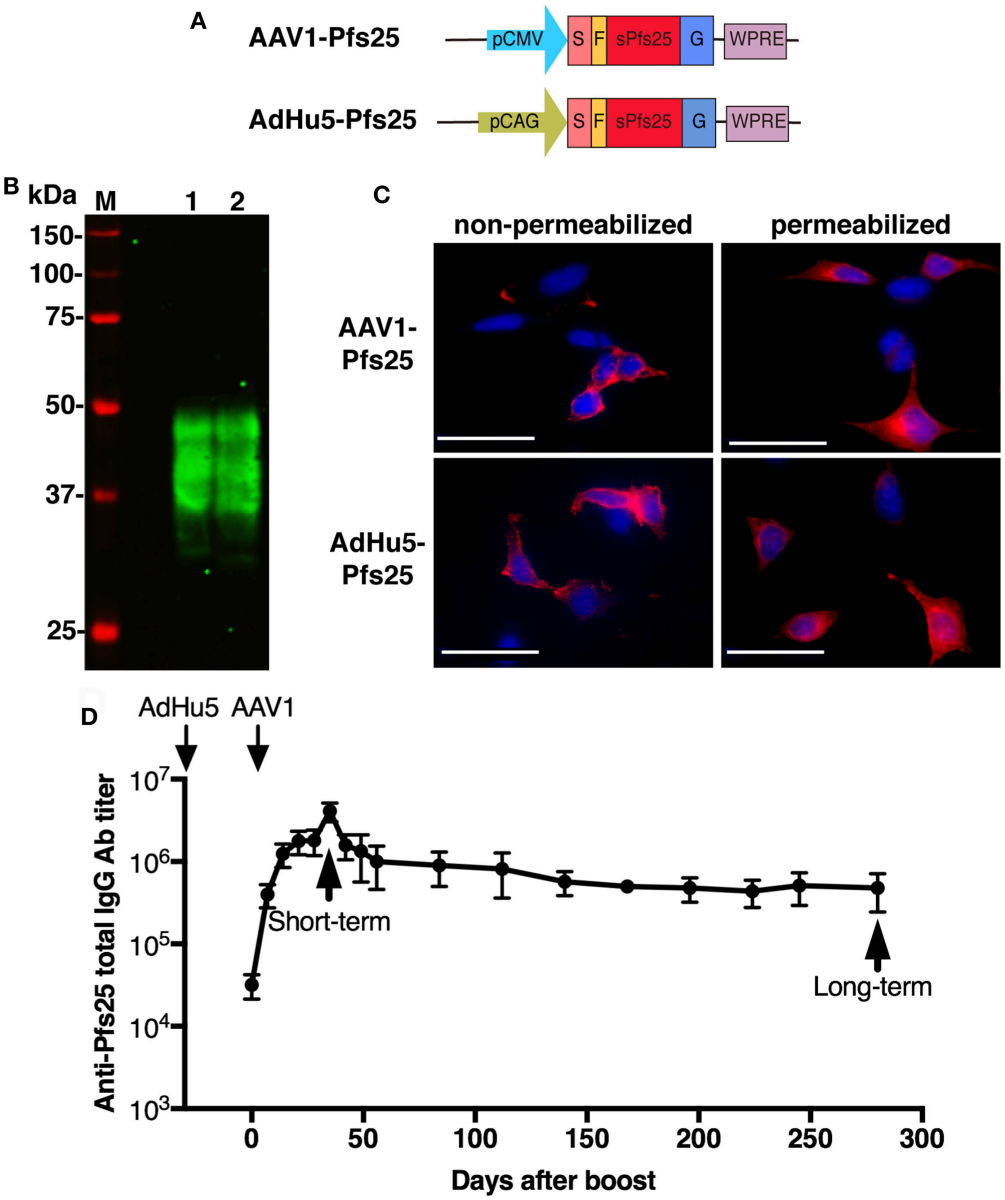
Functional activity of AdHu5-Pfs25 and AAV1-Pfs25. **(A)** Constructs of AdHu5-Pfs25 and AAV1-Pfs25. Expression of the *pfs25* gene cassette in AAV1 and AdHu5 was driven by a CMV promoter and CAG promoter, respectively. **(B)** Expression of Pfs25 in HEK293T cells transduced with AdHu5-Pfs25 (lane 1, MOI = 10) or AAV1-Pfs25 (lane 2, MOI = 10^6^), as assessed by western blotting using anti-Pfs25 mAb 4B7. **(C)** Localization of Pfs25 expression in HEK293T cells transduced with AAV1-Pfs25 (MOI = 10^5^) and AdHu5-Pfs25 (MOI = 10), as determined by IFA using Alexa-Fluor-594-conjugated 4B7 (red). **(D)** Monitoring of the anti-Pfs25 IgG titer. BALB/c mice (*n* = 5–10) were immunized with the indicated regimens at a 6-week-interval. Serum samples were collected from each mouse at 1 day before boost and weekly after boost. Anti-Pfs25 IgG titers were determined by ELISA and monitored for 280 days after booster injection. Right and left down arrows indicate prime and boost injections, respectively.

The ability of the AdHu5-AAV1 regimen to induce durable high-titer antibodies led us to further explore this immunization regimen for the development of a TBV. With this aim, we generated AAV1-Pfs25 and AdHu5-Pfs25 expressing the *pfs25* gene cassette fused to the VSV-G sequence (Figure 4A), which share a similar construction with AAV1-PfCSP-G(+) and AdHu5-PfCSP, respectively. To examine the expression of conformationally dependent Pfs25 TB epitopes, Pfs25-VSV-G in HEK293T cells transduced with AAV1-Pfs25 or AdHu5-Pfs25 were analyzed by immunoblotting under non-reduced conditions using anti-Pfs25 mAb (4B7), which recognizes a conformation-dependent epitope of Pfs25 (37). In cells transduced with either virus, 4B7 mAb reacted with Pfs25-VSV-G, resulting in a ladder of bands with relative *Mr* of 33–48 kDa (Figure 4B, lanes 1 and 2). We hypothesize that these multiple bands may be due to post-translational modifications because there are two potential *N*-linked glycosylation sites in the predicted amino acid sequence of Pfs25-VSV-G. IFA analysis showed that Pfs25-VSV-G in cells infected with either virus was expressed not only in the cytoplasm but also on the surface of the cells (Figure 4C). These results suggest that the Pfs25-VSV-G on the surface of the infected cells might retain the three-dimensional structure of the native Pfs25 protein, which is essential for the induction of antibodies with TB functionality (38).

### The AdHu5-AAV1 Pfs25 Immunization Regimen Induces Potent and Durable Anti-Pfs25 Antibody Responses

Since AdHu5-AAV1 PfCSP could induce durable anti-PfCSP antibody responses for 280 days, we addressed whether AdHu5-AAV1 Pfs25 also possesses this critical characteristic of a TBV. Anti-Pfs25 responses in immunized mice were monitored for 280 days. Consistent with the response induced by AdHu5-AAV1 PfCSP, immunization with AdHu5-AAV1 Pfs25 similarly maintained high anti-Pfs25 IgG titers over 280 days (Figure 4D).

### The AdHu5-AAV1 Pfs25 Regimen Elicits a Durable TB Effect for 287 Days

**Figure 5:**
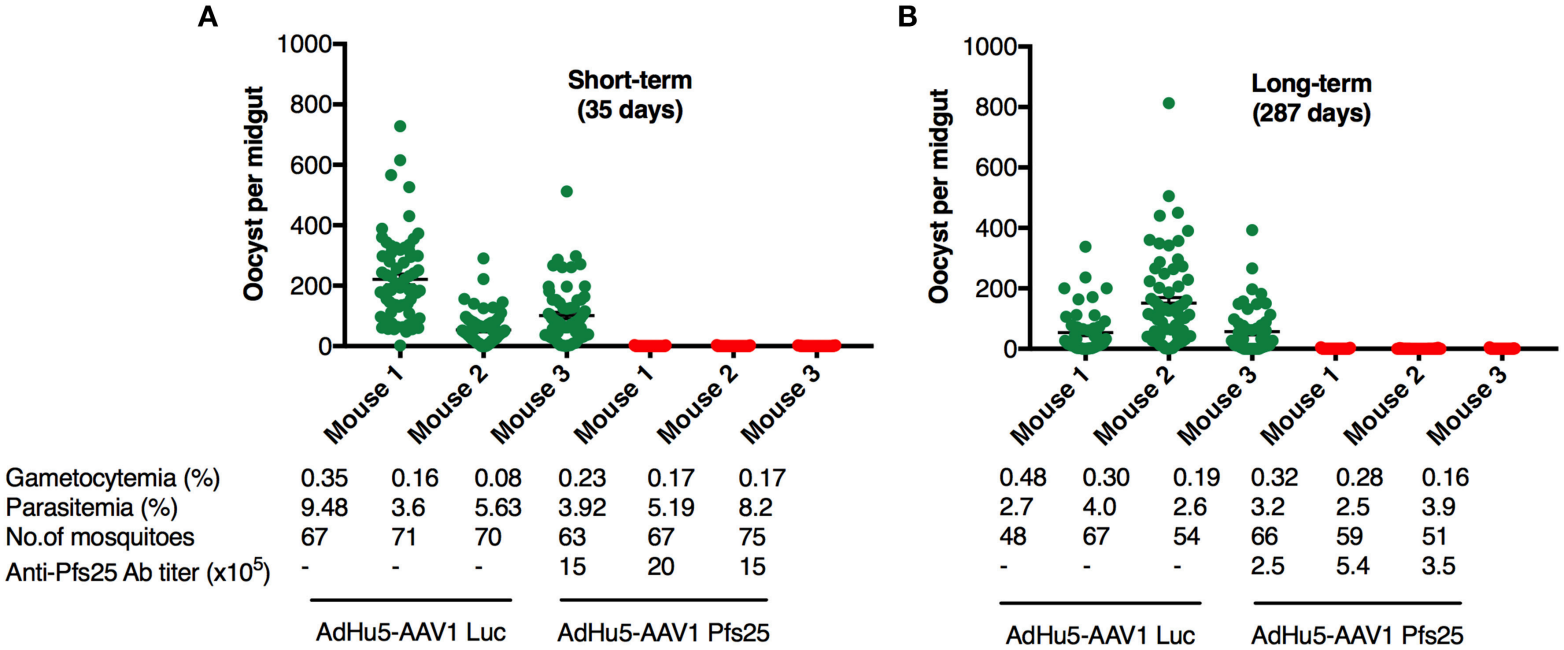
Transmission-blocking efficacy of the AdHu5-Pf25-prime and AAV1-Pfs25-boost regimen. **(A,B)** Groups of BALB/c mice (*n* = 3) were immunized with the indicated regimen at a 6-week interval and infected with Pfs25DR3 at 35 days **(A)** or 287 days **(B)** after boost. Mosquitoes were allowed to feed on the infected mice for a DFA. At day 10–12 post-feeding, the mosquito midguts were dissected, and oocyst intensity and prevalence were determined (Table 2). Each datapoint represents a single mosquito blood-fed on each mouse. The x-axis points represent individual mice. Horizontal lines indicate the mean numbers of oocysts observed (± standard errors of the means [SEM]).

It has been widely accepted that the TB efficacy relates directly to the anti-Pfs25 antibody titer (39). To evaluate the functional activity of the anti-Pfs25 antibody induced by our immunization regimen, we assessed the TB efficacies at 35 days (short-term) and 287 days (long-term) after boost by performing DFAs, which have been suggested to be about twice as effective at measuring the TB efficacy as the standard membrane-feeding assay (SMFA) (40). Groups of five mice each were infected i.p. with 10^6^ Pfs25DR3-pRBCs. At 3 days after infection, three of the five mice were chosen for DFA by parasitemia (>2%) and gametocytemia (>0.05%) (Figure 5). *A. stephensi* mosquitoes were allowed to feed on each infected mouse, and the oocyst intensity and prevalence were recorded at 10–12 days post-feeding. Reductions in the intensity and prevalence in the AdHu5-AAV1 Pfs25-immunized mice were calculated with respect to the AdHu5-AAV1-Luc-immunized controls.

A critical study suggested that, to obtain more reproducible data, TB assays should be analyzed only when controls have at least 35 oocysts per mosquito (41). In our short-term study, mosquitoes that fed on the three control mice displayed an average intensity of 125.17 oocysts/midgut, whereas those that fed on mice immunized with AdHu5-AAV1 Pfs25 had a mean intensity of 0.20 oocysts/midgut; thus, this immunization achieved a reduction (referred to as the TRA) of 99.84% (*p* < 0.0001). Correspondingly, the mean infection prevalence was reduced from 97.18 to 10.41%, achieving a significant reduction (referred to as the TBA) of 89.28% (*p* < 0.0001) (Table 2; Figure 5A).

**Table 2:** Transmission-blocking activity of the AdHu5-Pfs25-prime/AAV1-Pfs25-boost immunization regimen.

Group	Mean intensity ± SEM (oocysts per midgut)	Mean prevalence ± SEM (% infected mosquitoes)	TRA (%)^a^	TBA (%)^b^
**SHORT-TERM (35 DAYS)**				
AdHu5-AAV1 Pfs25	0.20 ± 0.04	10.41 ± 3.51	99.84^*^	89.28^*^
AdHu5-AAV1 Luc	125.17 ± 49.84	97.18 ± 2.15		
**LONG-TERM (287 DAYS)**				
AdHu5-AAV1 Pfs25	0.24 ± 0.03	12.72 ± 1.53	99.73^*^	85.97^*^
AdHu5-AAV1 Luc	87.24 ± 31.86	90.63 ± 5.67		

a Transmission-reducing activity (TRA) was calculated by comparison with the control group, and significant differences were assessed using a Mann–Whitney U-test (^*^p < 0.0001).

b *Transmission-blocking activity (TBA) was calculated by comparison with the control group, and significant differences were assessed using a Fisher's exact probability test (*

* *p < 0.0001)*.

In our long-term study, although the mean IgG titer was reduced to about 20% compared with that at day 35, the long-term TRA did not significantly decline over 287 days. Mosquitoes that fed on the three control mice displayed an average intensity of 87.24 oocysts/midgut, whereas the mean intensity following AdHu5-AAV1 Pfs25 immunization was only 0.24 oocysts/midgut, achieving a TRA of 99.72% (*p* < 0.0001). Correspondingly, the mean infection prevalence was reduced from 90.63 to 12.72%, achieving a significant TBA of 85.97% (*p* < 0.0001) (Table 2; Figure 5B).

Collectively, our data demonstrate that AAV1 is an excellent booster vaccine vector following an adenovirus prime; this regimen can induce a high level of humoral immune responses and achieve a high level of protective immunity and TB immunity against the malaria parasite.

## Discussion

In this study, we demonstrated durable PfCSP- and Pfs25-specific humoral responses elicited by a heterologous AdHu5-prime and AAV1-boost immunization regimen. We evaluated the efficacies of these immunizations by using transgenic *P. berghei* parasites expressing either PfCSP or Pfs25 in a murine model, which facilitates the optimization of vaccine immunogenicity *in vivo* (42). The regimen targeting the pre-erythrocytic stage antigen PfCSP elicited a high level of complete protection against sporozoite challenge. In the same way, the regimen targeting the mosquito-stage antigen Pfs25 conferred excellent TB activity as assessed using DFAs. Most notably, the TB activity was maintained up to 287 days after booster injection, with a TRA of 99%, fulfilling the requirement for an ideal TBV. Thus, AAV-based booster vaccines possess the remarkable characteristic of inducing durable antibody responses to major malaria vaccine candidate antigens following their administration after an AdHu5-priming vaccine, supporting the further evaluation of this regimen in clinical trials as a next-generation malaria vaccine platform.

It has been suggested that anti-CSP antibody titers are surrogate markers of protection for the magnitude and duration of RTS,S/AS01 efficacy (43). Waning anti-CSP antibody titers predict the duration of efficacy against clinical malaria. RTS,S/AS01 was shown to have a reduced efficacy from 36.3 to 4.45% over a 7-year follow-up (2, 3). Therefore, to achieve more durable protective efficacy, it is necessary to develop vaccines capable of inducing persistent anti-CSP antibodies. Here, we showed that using an AAV1 boost after an adenovirus prime evoked a long-term high titer of anti-PfCSP antibody. In addition, the regimen consists of only two doses, in contrast with the three doses needed for RTS,S; this reduced requirement will improve overall adherence to the immunization schedule.

Regarding the use of this modality as a TBV, we demonstrated that the two-dose regimen of AdHu5-AAV1 Pfs25 elicited durable anti-Pfs25 antibody with a high level of TB efficacy over 287 days after booster injection. Pfs25, a 25-kDa surface antigen of zygotes and ookinetes, is currently the most developed TBV candidate that has been tested in human clinical trials (44). However, using this antigen for the development of TBVs is challenging, as the antibody titer cannot be boosted by natural infection (6, 7). Furthermore, a high concentration of anti-Pfs25 IgG is required to achieve significant blocking activity (44). Cheru et al. found that the antibody concentration needed to reduce the number of oocysts by 50% in a SMFA was 85.6 μg/mL (45). Conjugation of Pfs25 to *Pseudomonas aeruginosa* exoprotein A (EPA) with Alhydrogel (46) improved the immunogenicity of the vaccine and induced a geometric mean of 88 μg/mL of anti-Pfs25 antibody in the highest dose group at 2 weeks after the fourth immunization in a Phase I trial (47). However, the antibody levels declined to near baseline within 1 year of immunization. Because the TBA of anti-Pfs25 correlates with the antibody titer, it is necessary for a TBV candidate to induce a durable high titer of anti-Pfs25 IgG. In the present study, our AdHu5-AAV1 Pfs25 immunization regimen achieved this highly desirable attribute.

An earlier AAV-based malaria vaccine candidate failed to achieve a sufficient antibody titer for malaria protection, even when the AAV was used for boosting after a prime with another AAV serotype or with DNA (19). Our strategy to combine an AAV-based vaccine with an adenovirus-based vaccine in a heterologous prime-boost regimen revealed that AAV has the potential to induce specific antibody against the encoded antigen, but only when it is administered as a boost, not as the prime. Although both adenovirus and AAV have been shown to be safe in human trials (10, 48), a potential area of concern in their application as vaccine vehicles is the pre-existing immunity in the human population due to previous exposure from natural infections, particularly adenovirus infections. It has been reported that the transgene product-specific antibody response was completely inhibited in humans after the administration of an AdHu5 vaccine vector (49), even with moderate titers of pre-existing neutralizing antibodies against AdHu5. A high prevalence of AdHu5-specific neutralizing antibodies was detected in both Gambian (84.67%) and South African (79.87%) populations (50). Nonetheless, in a phase 2 trial of AdHu5 vector-based Ebola vaccine in Sierra Leone, it was shown that a vaccine dose of 8.0 × 10^10^ viral particles was safe and highly immunogenic in healthy Sierra Leonean adults, inducing specific antibody responses from day 14 onwards, which peaked at day 28, but declined quickly in the following months (51). Thus, to maintain antibody responses against the transgene, we have shown that an AAV1 boost might be a solution. AAV1 has lower seroprevalence compared with AAV2, the prototype of AAV (52). Moreover, it has minimal cross-reactivity with pre-existing neutralizing antibodies against AAV2 (53). The seroprevalence of other AAV serotypes, such as AAV5, AAV6, or AAV8, is even lower; thus, the development of malaria vaccines based on other AAV serotypes is an exciting future goal (54).

In this study, the high level and durability of the TB efficacy conferred by the AdHu5-AAV1 Pfs25 immunization regimen is evidently correlated with the induction of a durable high titer of anti-Pfs25 total IgG. However, the protective efficacy against sporozoites was not correlated with the anti-PfCSP IgG level, and the current evaluation system did not address the correlation between protection and the cellular immune response. To identify the mechanism of protection elicited by the AdHu5-AAV1 PfCSP regimen, further experiments, particularly a cellular response analysis using isolated peripheral blood mononuclear cells, will be needed to clarify the cell-mediated immunity between protected and non-protected mice. Further studies are needed to evaluate the long-term cellular immunity and protection conferred by this regimen.

In the present study, the protective efficacy was evaluated using BALB/c mice. It is possible that the protection conferred by the AdHu5-AAV1 PfCSP occurred because the mice could induce CD8^+^ T cell responses to the *H-2K*^*d*^-restricted CD8^+^ T cell epitope that is encoded in the *pfcsp* gene of the transgenic PfCSP-Tc/Pb parasites (55). However, our previous study showed that although a homologous immunization regimen with AdHu5-PfCSP consistently induced higher CD8^+^ T cell responses compared with all other tested regimens, as evaluated by ELISpot and ICS assays, immunization with AdHu5-PfCSP alone failed to elicit high levels of protection (25). Importantly, heterologous prime-boost immunization regimens with AdHu5-PfCSP and our other viral-vectored vaccines are still effective against sporozoite intravenous challenge as well as against mosquito biting challenge (25), indicating that vaccine-induced cellular immune responses also play critical roles in protection. To improve the AdHu5-AAV1 vaccine system, it will be important to identify surrogate markers (cellular and humoral effectors) for protective efficacy. For this aim, animal experiments using other strains of mice and outbred mice are underway.

In conclusion, AAV1 is a potential viral vector for PfCSP and Pfs25 as a booster vaccine following an adenovirus prime. With a 99% TRA, the AdHu5-AAV1 Pfs25 immunization regimen appears to be a promising tool for achieving malaria eradication, as it has been shown that even a TRA of only 32% could reduce the basic reproduction number of the malarial parasite by 20% and eliminate *Plasmodium* from mosquito and mouse populations at low transmission intensities in a laboratory model (56). Thus, this immunization regimen deserves further evaluation in clinical trials, where it can be used without safety concerns because adenovirus and AAV have been previously applied in humans as vaccine and gene therapy vectors, respectively.

## Ethics Statement

All animal care and handling procedures were approved by the Animal Care and Use Committee of Kanazawa University (No. AP-163700) and in accordance with the Guidelines for Animal Care and Use prepared by Jichi Medical University (No. 09193). All efforts were made to minimize suffering in the animals.

## Author Contributions

YY, MI, and SY: study concept and design; YY, TY, MI, KY, SF, DY, AA, TE, FA, AI, HO, and SY: acquisition of data; YY, TY, MI, KY, and SY: analyses and interpretation of data; YY, MI, and SY: drafting the manuscript; MI and SY: critical revision of the manuscript for important intellectual content; YY, TY, and KY: statistical analyses; YY, TY, MI, KY, HM, SF, DY, AA, TE, FA, AI, ET, TT, HO, and SY: technical or material support; SY: study supervision.

### Conflict of Interest Statement

SY, MI, and HM are named inventors on filed patents related to immunization with the AAV anti-malaria vaccines. These products have not been commercialized. The remaining authors declare that the research was conducted in the absence of any commercial or financial relationships that could be construed as a potential conflict of interest.
