# Corrigendum: Adeno-associated virus as an effective malaria booster vaccine following adenovirus priming

**DOI:** 10.3389/fimmu.2025.1592118

**Published:** 2025-04-28

**Authors:** Yenni Yusuf, Tatsuya Yoshii, Mitsuhiro Iyori, Kunitaka Yoshida, Hiroaki Mizukami, Shinya Fukumoto, Daisuke S. Yamamoto, Asrar Alam, Talha Bin Emran, Fitri Amelia, Ashekul Islam, Hiromu Otsuka, Eizo Takashima, Takafumi Tsuboi, Shigeto Yoshida

**Affiliations:** ^1^ Laboratory of Vaccinology and Applied Immunology, Kanazawa University School of Pharmacy, Kanazawa University, Kanazawa, Japan; ^2^ Department of Parasitology, Faculty of Medicine, University of Hasanuddin, Makassar, Indonesia; ^3^ Kanazawa University Graduate School of Medical Sciences, Kanazawa University, Kanazawa, Japan; ^4^ Division of Gene therapy, Jichi Medical University, Shimotsuke, Japan; ^5^ National Research Centre for Protozoan Diseases, Obihiro University of Agriculture and Veterinary Medicine, Obihiro, Japan; ^6^ Division of Medical Zoology, Department of Infection and Immunity, Jichi Medical University, Shimotsuke, Japan; ^7^ Division of Malaria Research, Proteo-Science Center, Ehime University, Matsuyama, Japan

**Keywords:** *Plasmodium falciparum* circumsporozoite protein, Pfs25, human adenovirus serotype 5, adeno-associated virus, malaria vaccine, transmission-blocking

In the published article, there was an error in **Figure 3** as published. In the **Figure 3C**, the image of the non-permeabilized AdHu5-PfCSP is a duplicate of the non-permeabilized AAV1-PfCSP-G(+) image. The corrected **Figure 3** and its caption appear below.

**Figure 3:**
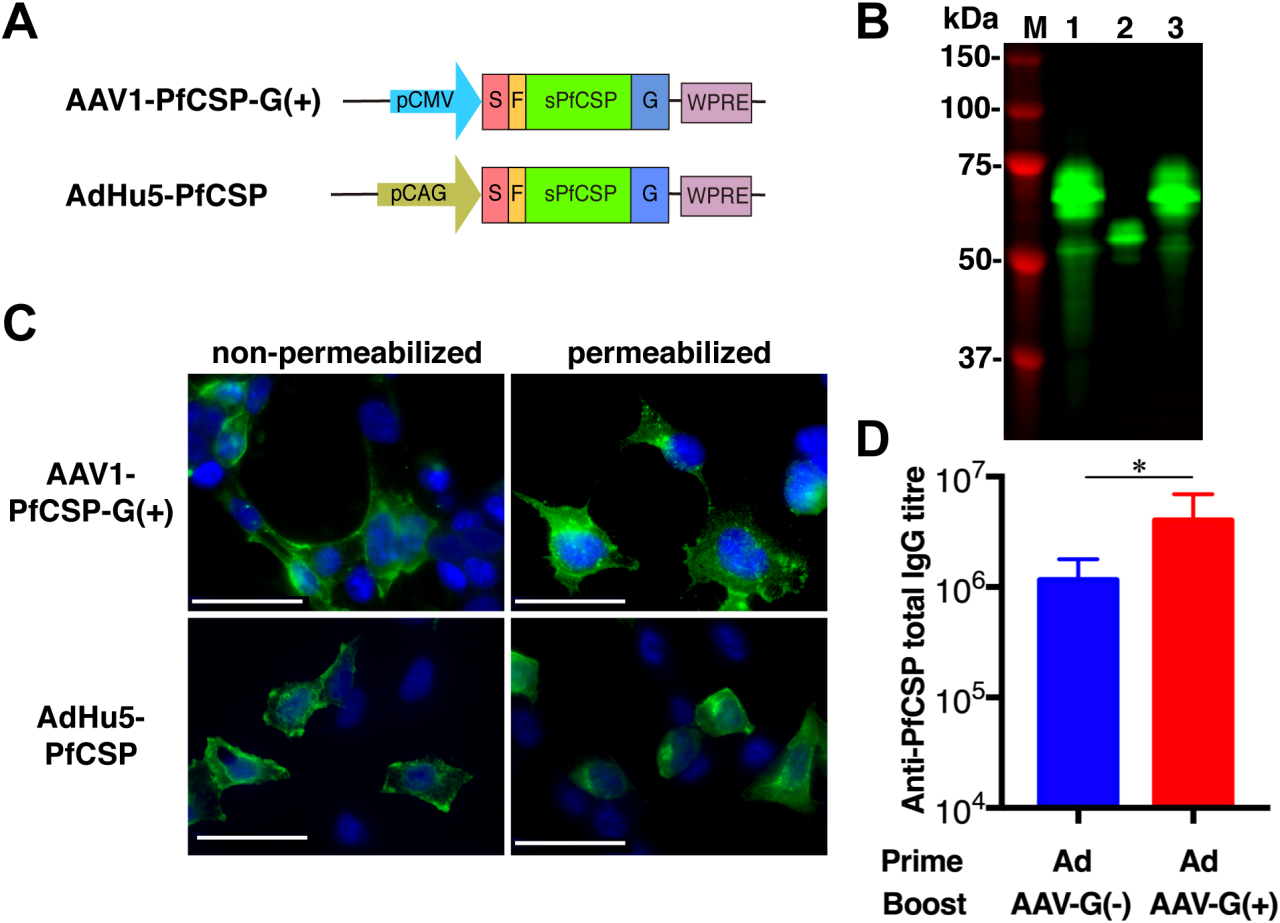
Functional activity of AAV1-PfCSP-G(+). **(A)** Constructs of AAV1-PfCSP-G(+) and AdHu5-PfCSP-G(+). Expression of the *pcsp* gene cassette in AAV1 and AdHu5 was driven by a CMV promoter and CAG promoter, respectively. G, VSV-G. **(B)** Expression of PfCSP in HEK293T cells transduced with AdHu5-PfCSP (lane 1, MOI = 3), AAV1-PfCSP-G(–) (lane 2, MOI = 10^5^), or AAV1-PfCSP-G(+) (lane 3, MOI = 10^5^), as assessed by immunoblotting with mAb 2A10 at 48 h post-transduction. **(C)** Localization of PfCSP expression in HEK293T cells transduced with AAV1-PfCSP-G(+) (MOI = 10^5^) and AdHu5-PfCSP (MOI = 10), as determined by IFA conducted as described in **Figure 2C**. **(D)** Anti-PfCSP IgG antibody responses. Groups of BALB/c mice (*n* = 10) were immunized with the indicated regimen at a 6-week interval. At 4 weeks post-boost, serum samples were collected from each mouse, and their anti-PfCSP IgG titers were determined by ELISA. AdHu5-PfCSP, AAV1-PfCSP-G(–), and AAV1-PfCSP (G+) are shown as AdHu5, AAV1-G(–), and AAV1-G(+), respectively. Bars and error bars indicate the means and SD of the values, respectively. Between-group differences were assessed with a Mann–Whitney *U*-test (*p < 0.05).

The authors apologize for this error and state that this does not change the scientific conclusions of the article in any way. The original article has been updated.

