# Supplementary figures and images for: Adeno-Associated Virus as an Effective Malaria Booster Vaccine Following Adenovirus Priming
Source: Front Immunol. 2019 Apr 5;10:730. doi: 10.3389/fimmu.2019.00730 (PMC6460511; doi:10.3389/fimmu.2019.00730)

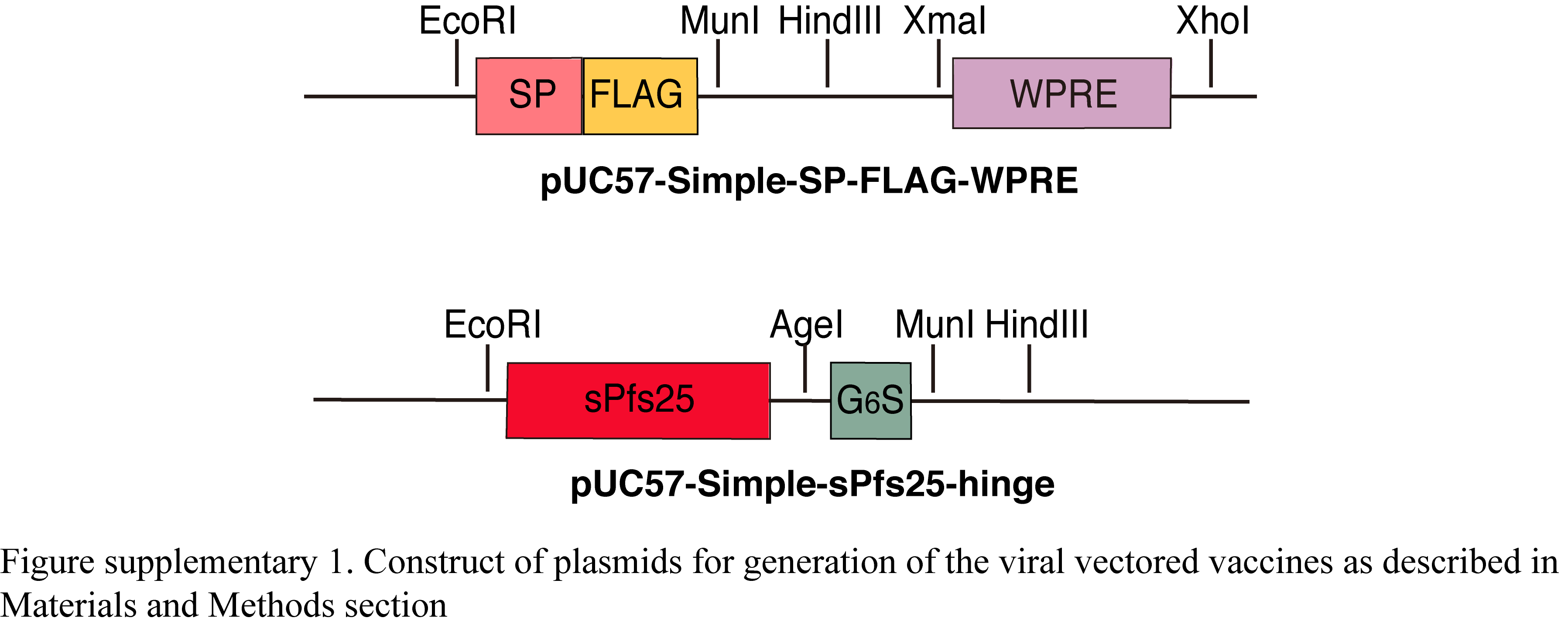

Supplement: Supplementary file 1 [file Image1.tif]

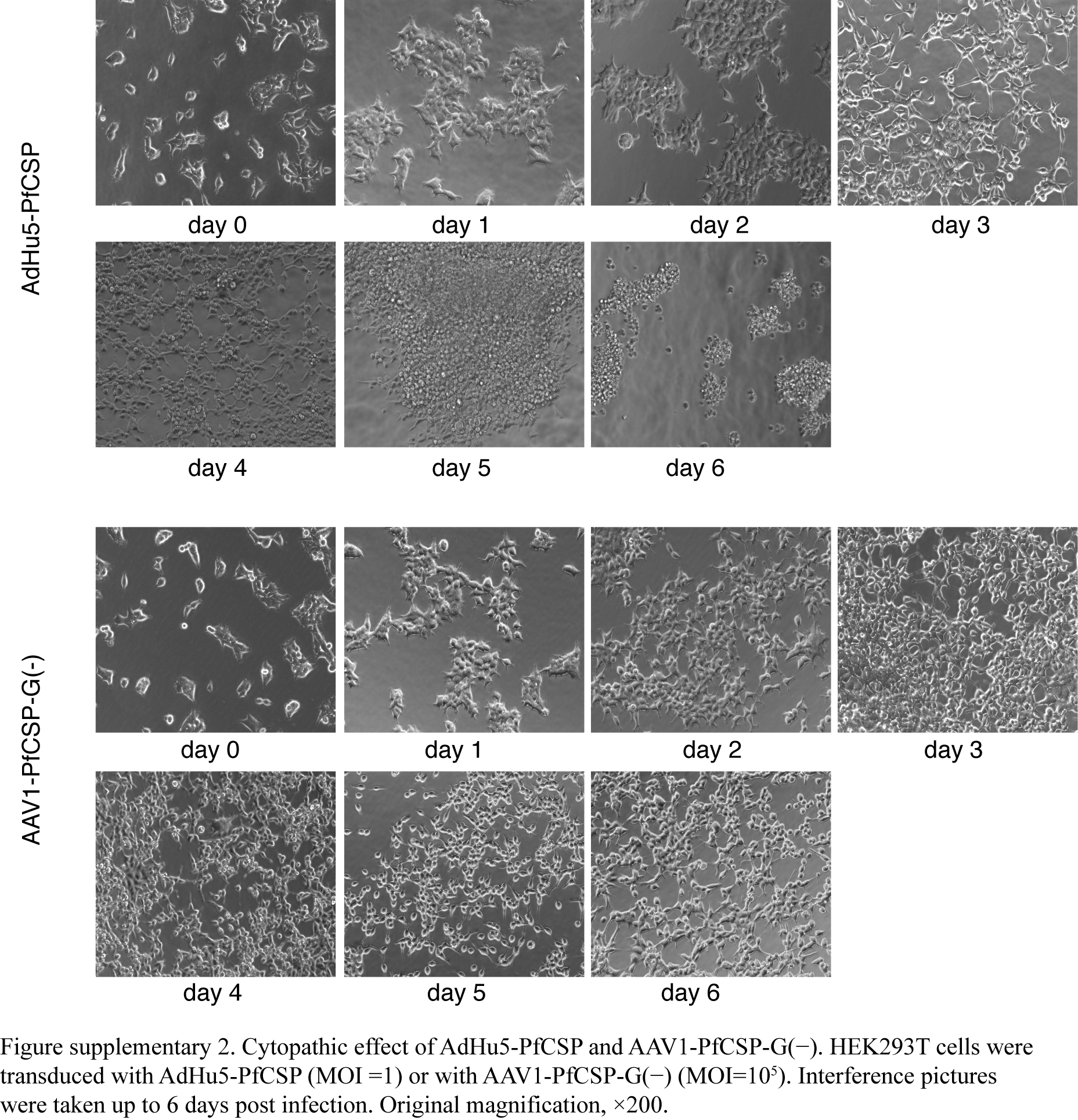

Supplement: Supplementary file 2 [file Image2.tif]
